# HDAnalyzeR: streamlining data analysis for biomarker research

**DOI:** 10.1093/bioadv/vbag020

**Published:** 2026-01-23

**Authors:** Konstantinos Antonopoulos, Emil Johansson, Josefin Kenrick, Leo Dahl, Fredrik Edfors, Mathias Uhlén, María Bueno Álvez

**Affiliations:** Department of Protein Science, SciLifeLab, KTH Royal Institute of Technology, Stockholm 17165, Sweden; Department of Protein Science, SciLifeLab, KTH Royal Institute of Technology, Stockholm 17165, Sweden; Department of Protein Science, SciLifeLab, KTH Royal Institute of Technology, Stockholm 17165, Sweden; Department of Protein Science, SciLifeLab, KTH Royal Institute of Technology, Stockholm 17165, Sweden; Department of Protein Science, SciLifeLab, KTH Royal Institute of Technology, Stockholm 17165, Sweden; Department of Protein Science, SciLifeLab, KTH Royal Institute of Technology, Stockholm 17165, Sweden; Department of Neuroscience, Karolinska Institutet, Stockholm 17165, Sweden; Department of Protein Science, SciLifeLab, KTH Royal Institute of Technology, Stockholm 17165, Sweden

## Abstract

**Motivation:**

Exploration of large-scale biological datasets remains a central challenge in computational biology. While many tools are available, they are often developed in isolation, leading to fragmented workflows, duplicated efforts, and limited reproducibility. There is a pressing need for flexible, standardized solutions that unify exploratory data analysis and biomarker discovery across diverse platforms.

**Results:**

We present HDAnalyzeR, a user-friendly and extensible R package for the streamlined analysis of high-dimensional biological data. HDAnalyzeR provides modular, reproducible workflows that support a range of analyses, from quality control and dimensionality reduction to differential expression and enrichment analysis. The package features built-in visualization, metadata-aware modeling, and seamless integration with interactive apps and learning resources. We also present two case studies, where HDAnalyzeR dramatically reduced analysis time and code complexity while providing biologically meaningful insights, such as classification of blood cancer types with AUC = 1.0 and identification of thousands of solid tumor-associated genes. HDAnalyzeR is designed to support both beginner users and experienced bioinformaticians, promoting transparency, reproducibility, and publication-quality output.

**Availability and implementation:**

HDAnalyzeR is freely available both as an open-source R package at https://github.com/kantonopoulos/HDAnalyzeR and a web application at https://hdanalyzer.serve.scilifelab.se.

## 1. Introduction

The increasing availability of large-scale biological datasets, including transcriptomics and proteomics, offers unprecedented opportunities to investigate complex biological questions. While these datasets are rich in information, their effective exploration is not trivial and, in several cases, remains a challenge (Li and Chen 2014, Morabito *et al.* 2025). One reason for this is the lack of consensus on how to systematically explore these datasets, particularly when it comes to identifying which genes, proteins, or other molecular features drive biological differences across conditions. Although numerous tools exist (Langfelder and Horvath 2008, Ritchie *et al.* 2015, Wu *et al.* 2021, Hao *et al.* 2024), they are often developed independently, requiring users to manually customize their workflows to combine multiple approaches. As a result, similar pipelines are frequently recreated, even within the same research group, leading to inefficiencies and a lack of standardization (Perkel 2019).

Here, we present HDAnalyzeR, a package developed within the context of the Human Disease Blood Atlas (HDBA) to support exploratory analyses and feature discovery in high-dimensional omics datasets. The HDBA is a comprehensive resource from the Human Protein Atlas initiative that maps protein expression profiles across blood samples from individuals with a variety of diseases (Álvez *et al.* 2025). While it was designed with a focus on biomarker discovery in proteomics data, the framework is adaptable to other data types, as demonstrated in a second case study using transcriptomics data from the Clinical Proteomic Tumor Analysis Consortium (CPTAC) (Edwards *et al.* 2015).

HDAnalyzeR promotes standardized, modular workflows for exploratory data analysis, differential expression, machine learning, and pathway enrichment. Our aim is to streamline the analytical process, reduce redundancy, and support good practices in data handling and interpretation. The package is equipped with a graphical user interface, publication-ready outputs, and extensive documentation, including tutorials and case studies, to support users of varying levels of expertise. By offering a unified, user-friendly platform for biomarker discovery, HDAnalyzeR helps researchers maximize the value of existing datasets while minimizing time spent on redundant pipeline development.

## 2 Methods

### 2.1 Data import and initialization

HDAnalyzeR includes multiple functions to facilitate efficient file handling. The “hd_import_data()” function supports the import of data in all common formats, while “hd_save_data()” enables saving data frames and R objects in various formats. Once the data and metadata are imported, they can be initialized with “hd_initialize(),” creating an HDAnalyzeR object that integrates seamlessly across all subsequent analyses. The input data can be in either long or wide format, with samples as rows and proteins, peptides, or genes as columns. Both data and metadata must contain a shared column of sample identifiers. Additionally, HDAnalyzeR provides functions for converting between long and wide formats, detecting variable types (continuous versus categorical), binning continuous variables, and applying log transformations.

### 2.2 Data preprocessing and exploratory data analysis

HDAnalyzeR offers a comprehensive suite of functions for exploratory data analysis (EDA). The “hd_qc_summary()” function performs an automated assessment, returning key metrics such as the number of rows and columns, proportions of categorical and continuous variables, missing value distributions, pairwise protein correlation heatmaps, and metadata variable distributions. If missing values are present, “hd_na_search()” provides a detailed visualization of their distribution across metadata categories, displaying percentage distributions as a heatmap.

For dimensionality reduction, HDAnalyzeR supports both Principal Component Analysis (PCA) and Uniform Manifold Approximation and Projection (UMAP) via *tidymodels* (version 1.2.0) (Kuhn and Wickham 2020). The “hd_auto_pca()” function performs PCA, returning component scores, a two-dimensional plot of user-defined components colored by a metadata variable, a loadings plot, and an explained variance plot. Similarly, “hd_auto_umap()” performs UMAP analysis, generating a two-dimensional representation of the data. If missing values are present, K-nearest neighbors (KNN) imputation (*k* = 5) is applied before analysis. This is used as a default as it offers a balanced trade-off between speed and accuracy, typically yielding reliable estimates for datasets with low-moderate levels of missingness.

Regarding preprocessing, HDAnalyzeR provides various imputation strategies. Median and KNN imputation are available via *tidymodels*, while an advanced nonparametric random forest-based imputation is implemented via *missForest* (version 1.5) (Stekhoven 2022). Missing values can alternatively be removed using “hd_omit_na(),” which allows selective removal of samples based on missing data. Normalization is performed using “hd_normalize(),” which supports z-score scaling and batch effect removal via *limma* (version 3.60.4) (Ritchie *et al.* 2015), as frequently used in omics datasets. The package also provides hierarchical clustering methods enabling predefined clustering, optimal cluster number determination via the gap statistic, and assessment of cluster stability using bootstrapping with Jaccard index calculations.

### 2.3 Differential expression analysis

HDAnalyzeR supports differential protein expression analysis via two statistical approaches. “hd_de_limma()” employs linear modeling with empirical Bayes moderation (*limma*) (Ritchie *et al.* 2015), allowing for the inclusion of metadata variable covariates to adjust for potential confounders. Alternatively, “hd_de_ttest()” applies a two-sided *t*-test for simpler comparisons. Both methods incorporate Benjamini-Hochberg correction for multiple hypothesis testing. Results can be visualized using “hd_plot_volcano(),” which generates volcano plots displaying fold changes and adjusted *P*-values. The samples containing missing values in either the case–control groups or the covariate variables will be removed prior to the analysis.

### 2.4 Predictive modeling

Predictive modeling pipelines in HDAnalyzeR are built using *tidymodels* (Kuhn and Wickham 2020). The “hd_split_data()” function partitions data into training and test sets, ensuring stratification based on case–control status. Regularized regression (“hd_model_rreg()”) and random forest (“hd_model_rf()”) pipelines support both classification and regression tasks. These pipelines employ cross-validation and Latin hypercube grid search to optimize hyperparameters before selecting the best-performing model based on Area Under the Curve (AUC) for classification and Root Mean Square Error (RMSE) for regression. The final model is refitted to the full training dataset and evaluated on the test set. Output metrics include accuracy, sensitivity, specificity, AUC, and confusion matrices for classification, as well as RMSE and R^2^ for regression. Visualizations include probability plots and feature importance bar plots (scaled 0–1), with permutation importance used for random forests. For random forest models, features with negative permutation importance are set to zero prior to scaling as they can be considered non-informative (Strobl *et al.* 2008). For both regularized regression and random forest, absolute coefficient values are scaled using min-max normalization, with the minimum fixed at zero to preserve all non-zero features. Additionally, for GLM-based models, the original sign of the coefficient is retained in the output to indicate the direction of the association. For multi-class classification, AUC calculations are performed using *multiROC* (version 1.1.1) (Wei and Wang 2018), reporting per-class AUC as well as macro- and micro-averaged AUC. When only one or two predictors are available, logistic regression is recommended instead (“hd_model_lr()”). Trained models can be validated on independent datasets using “hd_model_test(),” which retrains the model on the full discovery dataset before evaluating it on the external dataset. If missing values are present, KNN imputation (*k* = 5) is applied.

### 2.5 Co-expression network analysis

Co-expression network analysis is performed via “hd_wgcna()” using *WGCNA* (version 1.73) (Langfelder and Horvath 2008). The minimum sample size to run this type of analysis is 15 samples. The function constructs gene co-expression networks, with soft-thresholding power either predefined or optimized automatically aiming to create a network with a scale-free topology. The “hd_plot_wgcna()” function generates various visualizations, including protein clustering dendrograms, module-annotated co-expression heatmaps, module eigengene (ME) adjacency heatmaps, and predictive power score (PPS) heatmaps depicting associations between MEs and metadata variables. If missing values are present, KNN imputation (*k* = 5) is applied before network construction.

### 2.6 Pathway enrichment analysis and automated literature search

HDAnalyzeR supports pathway enrichment analysis using two approaches: Over-Representation Analysis (ORA) via “hd_ora()” and Gene Set Enrichment Analysis (GSEA) via “hd_gsea().” Both methods leverage *clusterProfiler* (version 4.12.3) (Wu *et al.* 2021), converting gene names to ENTREZ IDs for compatibility with annotation databases. Supported databases include Reactome (*ReactomePA* version 1.48.0) (Yu and He 2016), Kyoto Encyclopedia of Genes and Genomes (*KEGGREST* version 1.44.1) (Tenenbaum and Bioconductor Package Maintainer 2024), and Gene Ontology (*org.Hs.eg.db* version 3.19.1) (Carlson 2024) categories: biological process (BP), cellular component (CC), and molecular function (MF). Multiple testing correction is applied using the Benjamini-Hochberg method. To mitigate false positives, predefined background gene sets for various proteomics platforms can be used. Visualization options include dot plots, gene-concept networks, and hierarchical clustering (tree plots), generated via “hd_plot_ora()” and “hd_gsea().”

Automated literature searches are conducted via “hd_literature_search()” using *easyPubMed* (version 2.13) (Fantini 2019). This function constructs queries in the format “Protein AND Disease AND Keywords”, retrieves relevant publications from NCBI PubMed, and returns a structured table of results.

### 2.7 Data visualization

Data visualization in HDAnalyzeR is primarily implemented using *ggplot2* (version 3.5.1) (Wickham 2016), supplemented by *ggridges* (version 0.5.6) (Wilke 2024), *ggraph* (version 2.2.1) (Pedersen 2024), *ggrepel* (version 0.9.6) (Slowikowski 2024), and *tidyheatmaps* (version 0.2.1) (Engler 2024). The package includes a custom *ggplot2* theme, color palettes, and additional palettes from *ggsci* (version 3.2.0) (Xiao 2024) to ensure visually consistent and publication-quality figures.

### 2.8 HDAnalyzeR web application

The HDAnalyzeR web application was developed with *shiny* (version 1.9.1) (Chang *et al.* 2024) and serves as an intuitive interface for users with limited coding experience, providing seamless access to the package’s functionalities. Users can upload their data and metadata following the same formatting requirements as the R package. The maximum file size the user can upload is 500 MB. The application enables exploratory data analysis through interactive visualizations, including scatter plots, box plots, bar plots, and histograms, which can be generated by selecting variables. Additionally, users can filter specific samples or features and remove missing values. If missing values are not removed, they are automatically imputed using KNN (*k* = 5) for dimensionality reduction and machine learning models. The app is available at: https://hdanalyzer.serve.scilifelab.se.

The web app supports PCA and UMAP for dimensionality reduction, differential expression analysis via *limma*, and machine learning classification or regression using the LASSO algorithm. All analyses are executed through an intuitive point-and-click interface, guiding users through each step of the workflow. The results are returned as tables the user can download in CSV format or as interactive visualizations, powered by *plotly* (version 4.10.4) (Sievert 2020), ensuring an accessible and user-friendly experience.

## 3 Results

### 3.1 HDAnalyzeR overview

HDAnalyzeR is designed to streamline common proteomics analysis workflows for biomarker discovery. While optimized for Olink proteomics datasets, it is compatible with other proteomics platforms such as mass spectrometry, Luminex, SomaScan, and Alamar, as well as other omics data types, including transcriptomics. The package functions should be reviewed to determine the best approach for their specific dataset. Table 1 provides a summary of the requirements the data should have before utilizing HDAnalyzeR.

**Table 1:** General requirements.

Requirement	Description
Data	Long format requires at least three columns: sample/ID, feature (e.g. proteins or genes) and measurement (e.g. TPM, NPX, RFU). Wide format requires the following structure: Rows = samples; Columns = sample/ID (first column) + features.
Metadata	Table with one row per sample; includes sample/ID and any required columns (e.g. group labels, covariates).
Standardized feature IDs	Recommended to use gene symbols, UniProt IDs, etc. depending on platform. Feature IDs may be platform-specific, mapping tables can help translate to gene symbols when required (e.g. pathway analyses).
Matching sample IDs	Sample IDs must match exactly between metadata and data matrix.

**Figure 1:**
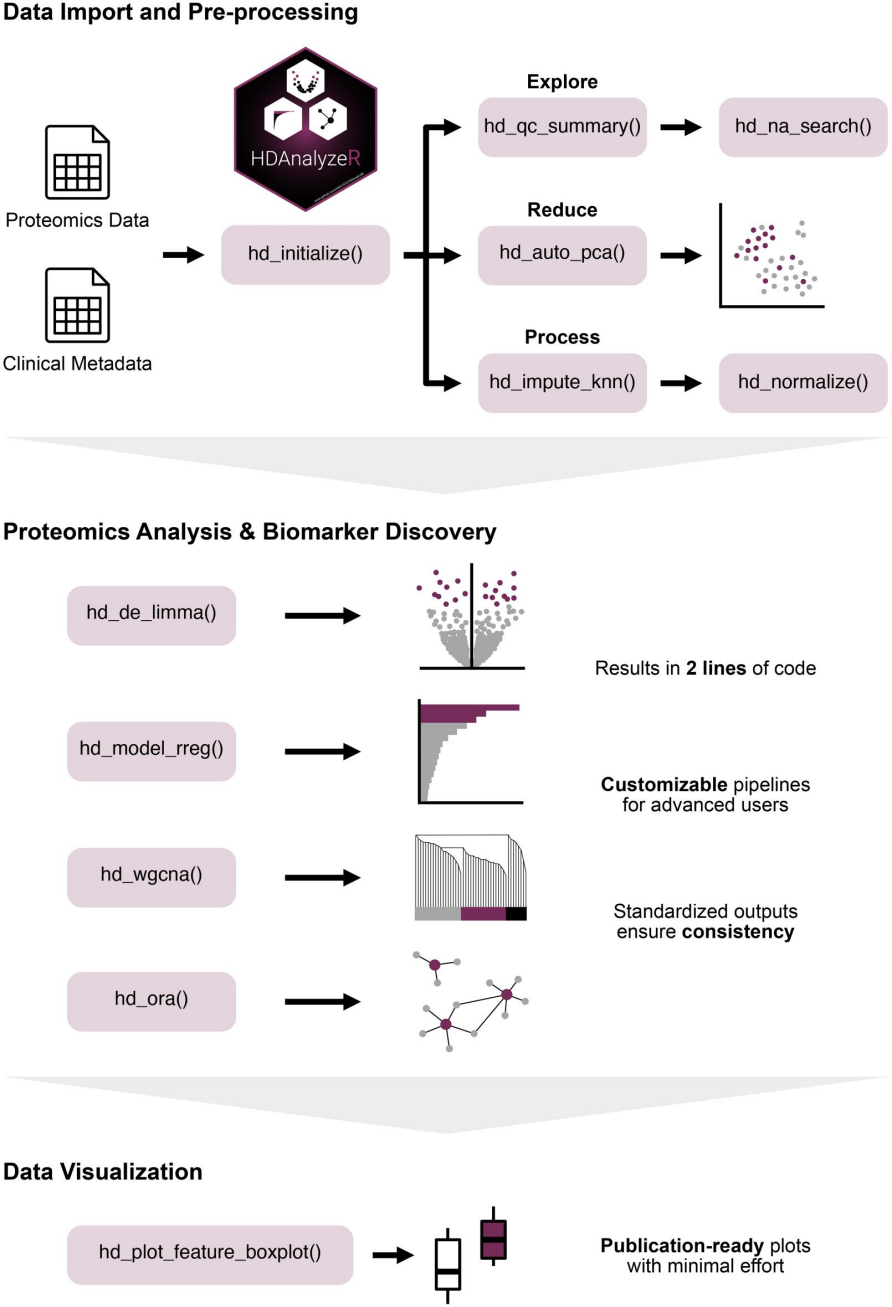
HDAnalyzeR Workflow. HDAnalyzeR streamlines proteomics data analysis by providing functions for data import, preprocessing, core analytical workflows, and result visualization. The functions are categorized into three main modules: (ⅰ) Data Import & Preprocessing, which handles data exploration and formatting, quality control, imputation, and normalization; (ⅱ) Proteomics Analysis & Biomarker Discovery, encompassing differential expression analysis, machine learning, co-expression network analysis, and pathway enrichment; and (ⅲ) Data Visualization, offering publication-ready plots for enhanced interpretability and communication of results.

The package facilitates the entire analytical pipeline, from data import and exploratory analysis to preprocessing, differential expression analysis, machine learning-based classification, gene co-expression analysis, and pathway enrichment. At every step, HDAnalyzeR generates insights and high-quality, publication-ready visualizations. A schematic representation of the package structure is presented in Fig. 1. In comparison with widely used R/Bioconductor tools, HDAnalyzeR is the only package that integrates all major analysis components into a single, coherent and modular framework (Table 1, available as supplementary data at *Bioinformatics Advances* online).

Our goal was to develop a tool that balances ease of use with analytical flexibility. HDAnalyzeR enables users to execute complex proteomics workflows through simple, intuitive function calls while retaining customization options for more advanced users. Comprehensive documentation and example guides are available on the HDAnalyzeR website, detailing individual functions and complete analytical workflows. Most of the exported results and visualizations are returned as modifiable objects, allowing further customization and integration into downstream analyses.

To demonstrate the versatility and practical applications of HDAnalyzeR, we present two illustrative case studies. Each showcases the analysis of publicly available datasets generated using two widely adopted proteomics platforms. The examples represent more complex workflows, integrating multiple analytical steps into a complete pipeline. For users interested in simpler applications and step-by-step examples, we refer to the HDAnalyzeR vignettes available at https://kantonopoulos.github.io/HDAnalyzeR/articles/. It is important to note that, for optimal use of the package, data should be preprocessed and fulfill specific requirements (Table 1).

### 3.2 Case study 1: plasma proteomics of hematological malignancies using proximity extension assay

To demonstrate the practical utility of HDAnalyzeR, we applied it to a subset of the U-CAN proteomics dataset presented by Álvez *et al.* (2023). The dataset was generated using the Olink Explore 1536 platform and includes diverse malignancies. For this case study, we focused on a subset of patients diagnosed with blood cancers, specifically, Acute Myeloid Leukemia (AML; *n* = 50), Chronic Lymphocytic Leukemia (CLL; *n* = 48), and Multiple Myeloma (MYEL; *n* = 38), with the aim of identifying a plasma protein panel capable of distinguishing CLL patients from the others in a biologically meaningful way.

After importing and subsetting the dataset, we initialized the HDAnalyzeR object and split the data into training and validation sets. All analysis steps, including feature selection and model tuning, were conducted exclusively on the training set to prevent information leakage and ensure robust model performance estimation. The held-out validation set was used only for final model evaluation.

**Figure 2:**
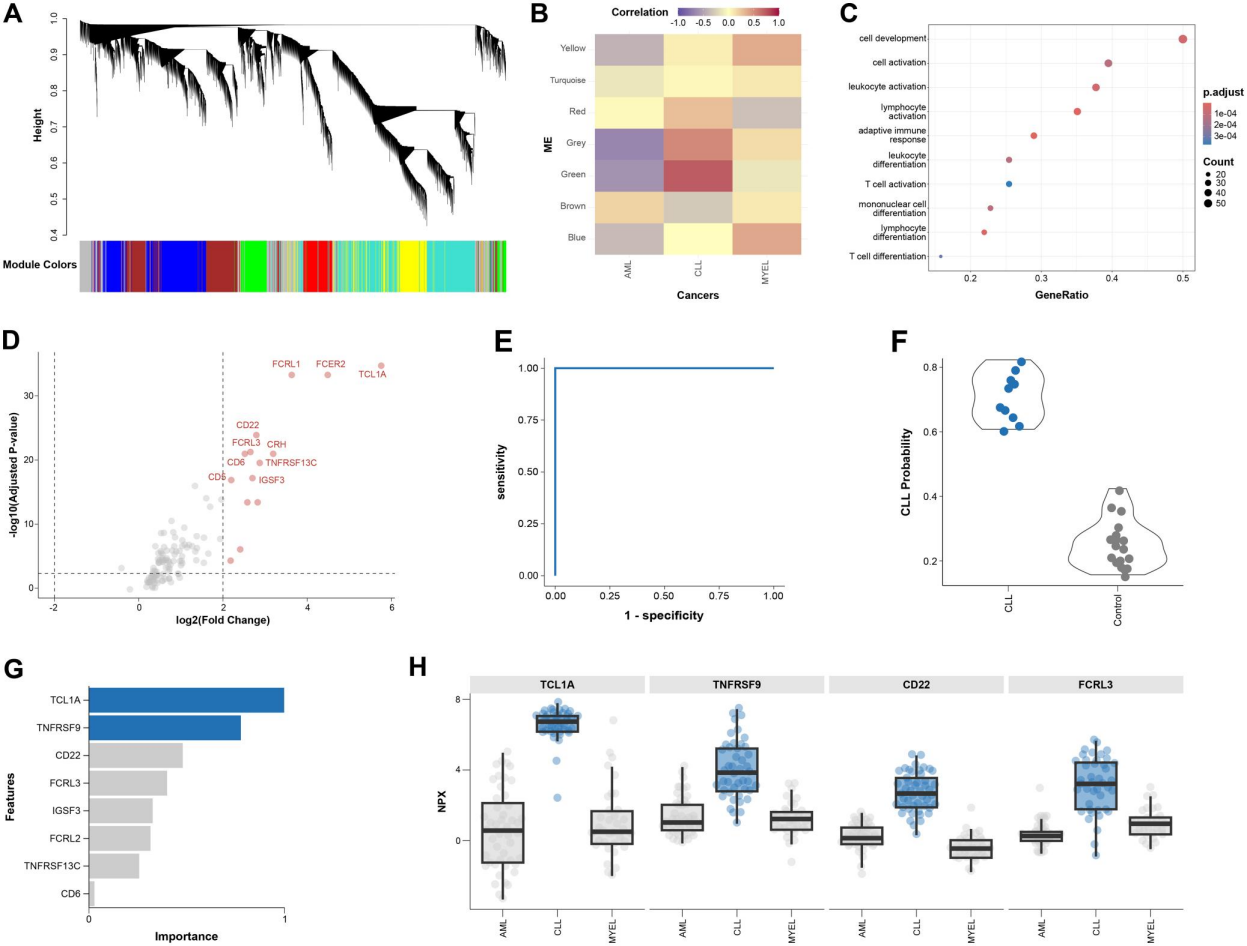
Identification of a protein biomarker panel for CLL using HDAnalyzeR. (A) Weighted gene co-expression network analysis (WGCNA) clustered proteins into seven modules: green (*n* = 117), red (*n* = 100), brown (*n* = 230), turquoise (*n* = 360), yellow (*n* = 159), blue (*n* = 260), and grey (*n* = 237; unassigned). (B) Module-trait correlation matrix showing Pearson correlations between each module eigengene and cancer status; the green module showed the strongest positive correlation with CLL (r = 0.73). (C) Pathway enrichment (ORA) of proteins in the green module highlights enrichment in immune-related and cell activation pathways. (D) Volcano plot of differential expression comparing CLL to AML+MYEL controls. Of the 117 proteins in the green module, 14 are significantly upregulated (|log_2_FC| ≥ 2, FDR-adjusted p ≤ 0.005). (E) Receiver operating characteristic (ROC) curve of the final model evaluated on the test set (AUC = 1.0). (F) Predicted class probabilities for CLL versus AML+MYEL control samples, showing clear separation. (G) Scaled feature importance (0–1) from the LASSO model trained on the top 10 proteins ranked by |kME| × log_2_FC; 8 features are retained, with 2 showing importance > 0.5. (H) Box plots of the top four predictive proteins in the final model, including TCL1A, illustrating strong differential expression between CLL and controls.

We first performed a weighted gene co-expression network analysis (WGCNA) to identify modules of proteins with similar expression patterns and assess their association with the CLL phenotype. This served as an initial step in feature selection. The analysis grouped the proteins into six distinct modules, in addition to the unassigned “grey” module (Fig. 2A). Among these, only the green module (*n* = 117 proteins) exhibited a strong correlation with CLL status (Pearson’s *r* = 0.73; Fig. 2B), and we retained this module for downstream analysis.

To investigate the biological relevance of the green module, we performed pathway enrichment analysis using overrepresentation analysis (ORA). The results revealed that the module’s proteins are significantly enriched in pathways related to cell activation, immune cell differentiation, and lymphocyte-mediated immune responses (Fig. 2C), consistent with the pathophysiology of CLL, an immune cell-driven cancer.

Next, we applied differential expression analysis to further refine the feature set. We compared CLL samples against a combined control group of AML and MYEL patients, applying stringent thresholds (|log_2_FC| ≥ 2 and FDR-adjusted *P* ≤ .005). This yielded 14 significantly upregulated proteins out of the original 117 (Fig. 2D). To prioritize proteins with both strong differential expression and centrality within the co-expression network, we ranked these based on the product of |kME| (module membership) and log_2_ fold change, selecting the top 10 candidates.

These 10 proteins were then used to train a LASSO classification model to predict CLL status. The model pipeline included class balancing, removal of highly correlated features (Pearson *r* > 0.9), and hyperparameter tuning using five-fold cross-validation and a Latin hypercube grid of 30 penalty values. On the held-out validation set, the final model achieved perfect classification performance (ROC AUC = 1.0; Fig. 2E) and predicted class probabilities showed clear separation between groups (Fig. 2F). The model retained 8 out of 10 proteins, with two proteins exhibiting scaled feature importance above 0.5 (Fig. 2G).

To further visualize discriminatory power, we plotted the expression distributions of the top four features, revealing clear expression differences, especially for *TCL1A*, which showed strong potential as a single-marker classifier (Fig. 2H).

Finally, using “hd_literature_search()”, we queried PubMed for the four top-ranked proteins in our predictive panel and confirmed their relevance to CLL. *TCL1A* is a well-established oncogene in CLL, with recent studies highlighting its role in leukemogenesis and immune microenvironment modulation (Jiang *et al.* 2023, Hayakawa *et al*. 2024, Pfeuffer *et al.* 2024). *TNFRSF9* (*4-1BB*) has been implicated in the immune dysregulation observed in CLL and has potential as an immunotherapeutic target (Ukrainskaya *et al.* 2023). *CD22*, a B-cell-specific surface molecule, has shown diagnostic and therapeutic relevance in CLL and is actively explored in antibody-drug conjugates and CAR-T approaches (Su *et al.* 2025). *FCRL3*, part of the Fc receptor-like family, is expressed in CLL and associated with altered immune signaling and disease progression (Klintman *et al.* 2021).

To assess the robustness of the analytical workflow and the influence of user-defined choices, we performed a targeted sensitivity analysis across three major stages of Case Study 1. Variation in KNN-imputation hyperparameters showed negligible impact, with changes in the number of neighbors producing variance <1 × 10^−4^ in the imputed values (Fig. 1A, available as supplementary data at *Bioinformatics Advances* online). In contrast, differential expression results were sensitive to statistical thresholds as relaxing the log_2_ fold-change or adjusted *P*-value cutoffs increased the number of detected proteins, consistent with expected behavior across significance filters (Fig. 1B, available as supplementary data at *Bioinformatics Advances* online). For the classification task, modifying the train/test split, cross-validation scheme or hyperparameter grid had no measurable effect on AUC or feature selection, reflecting the intrinsic separability of this dataset. To limit opportunities for outcome-hacking, HDAnalyzeR enforces established good practices, including mandatory train/test separation, a minimum of two cross-validation folds and the use of BH-adjusted *P*-values, and provides community established defaults that allow users to perform rapid analyses without compromising methodological integrity.

### 3.3 Case study 2: transcriptomic signatures across solid tumors in Clinical Proteomic Tumor Analysis Consortium datasets

To further showcase the versatility of HDAnalyzeR, we applied the tool to transcriptomics data from the CPTAC (Edwards *et al.* 2015). In this use case, we focused on three solid tumor types (kidney, lung, and endometrial cancers) to perform a conventional biomarker discovery analysis in a case–control setting, comparing tumor tissues with their matched adjacent normal counterparts.

The data, already normalized as transcripts per million (TPM), were log_2_-transformed using the expression $\log_{2}\left(\mathrm{TPM}+1\right)$ to reduce skewness and ensure comparability, particularly for low-expression genes. After parsing key metadata such as sample type, sex, and age, we initialized the HDAnalyzeR object and proceeded with the analysis.

**Figure 3:**
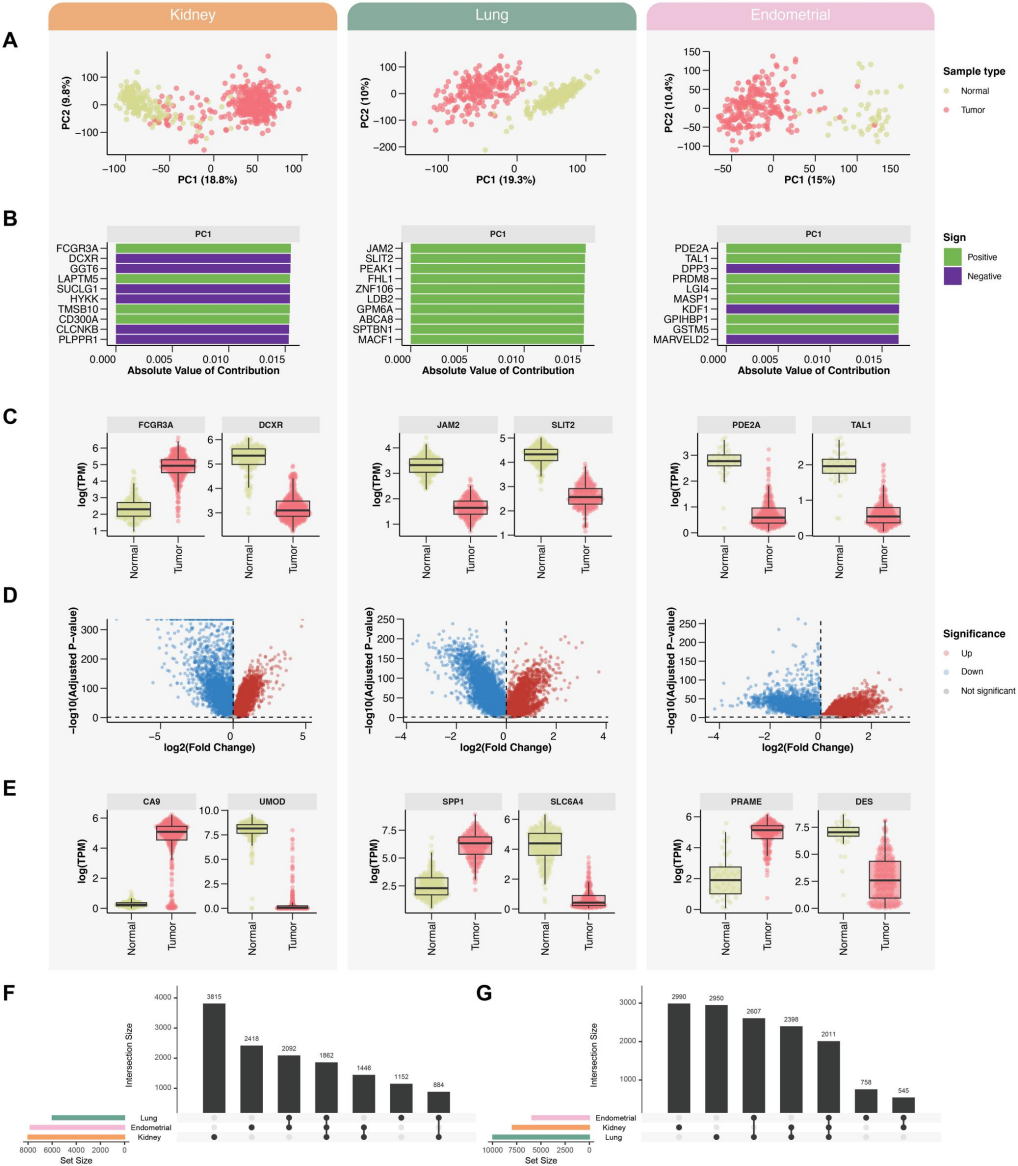
Transcriptomic biomarker discovery in three solid tumors using HDAnalyzeR. (A) Principal component analysis (PCA) of log_2_-transformed TPM data for kidney, lung, and endometrial cancer datasets. Tumor and adjacent normal tissues form distinct clusters along the first two principal components in each cancer type. (B) Top 10 gene contributors to the first two principal components across the three cancers. (C) Expression boxplots of the top two PCA-contributing genes per cancer type highlight strong separation between tumor and normal samples. (D) Volcano plots from differential expression analysis reveal significantly up- and downregulated genes in tumor versus normal tissue, adjusted for age and sex. (E) Expression boxplots for representative top markers in each cancer: *CA9* and *UMOD* (kidney), *SPP1* and *SLC6A4* (lung), *PRAME* and *DES* (endometrial). (F and G) UpSet plots summarizing overlaps among significantly upregulated (F) and downregulated (G) genes across the three cancer types. Despite some overlap, each tumor type displays a distinct transcriptional signature.

PCA was performed separately for each cancer type to evaluate data structure and identify potential outliers. In all three datasets, PCA revealed a clear separation between tumor and normal tissue samples in the PC1-PC2 plane, suggesting distinct transcriptomic profiles between the groups. The first two principal components accounted for approximately 30% of the total variance across all datasets (Fig. 3A), and no substantial outliers were detected. Analysis of the top 10 contributing genes to the principal components indicated cancer-type-specific patterns. In lung cancer, the top contributors exhibited uniformly positive loadings, while in kidney and endometrial cancers, the loadings were a mixture of positive and negative values (Fig. 3B). Expression boxplots of the top two PCA-contributing genes for each cancer further confirmed their ability to discriminate between tumor and normal samples (Fig. 3C). For example, in kidney cancer, *DCXR* was downregulated, suggesting a tumor-suppressive or protective role (Perco *et al.* 2019). In lung cancer, *JAM2* was downregulated, with reduced expression associated with increased tumor invasiveness, poorer prognosis, and diminished immune infiltration (Chen *et al.* 2025), whereas *SLIT2* appeared downregulated, supporting its known function as a tumor suppressor (Dallol *et al.* 2002). In endometrial cancer, *PDE2A* expression was markedly reduced, consistent with its reported role as a tumor suppressor in various cancer types including ovarian cancer (Yu et al. 2024). The trends reported for all listed examples were verified by the proteomics CPTAC data (Li et al., 2023), presented in the Human Protein Atlas (Uhlén et al., 2015).

We next performed differential expression analysis using the *limma* package in a tumor-versus-normal design, correcting for age and sex to control for confounding demographic effects. The resulting volcano plots revealed numerous significantly upregulated and downregulated genes across all three cancer types (Fig. 3D), with kidney cancer displaying the largest number of differentially expressed genes overall. Expression plots of the most up- and downregulated genes highlighted the clear separation between groups, particularly in kidney cancer, where the signal appeared strongest (Fig. 3E). Among the kidney markers, *CA9* emerged as a highly upregulated gene, consistent with its established role as a diagnostic and prognostic biomarker in clear cell renal cell carcinoma (Tostain *et al.* 2010). In contrast, *UMOD*, though kidney-specific and implicated in chronic kidney disease, showed more limited evidence for direct involvement in cancer (Zaucke *et al.* 2010, Trevisani *et al.* 2019). In lung cancer, *SPP1* was strongly upregulated and is well-known to contribute to tumor aggressiveness and immune modulation (Matsubara *et al.* 2022, Li *et al.* 2024). *SLC6A4*, a serotonin transporter, also showed expression changes, although its role in lung cancer remains underexplored (Tu *et al.* 2022). In endometrial cancer, *PRAME* was frequently expressed across both endometrioid and serous subtypes and is emerging as a candidate immunotherapy target (Coppock *et al*. 2023). These examples are supported by the proteomics CPTAC data presented in the Human Protein Atlas, except for PRAME, which shows the same trend in endometrial cancers with *P*-value < 8e-5 but does not reach the defined significance threshold.

To summarize and compare results across the three cancer types, we visualized the sets of upregulated and downregulated genes using UpSet plots (Fig. 3F and G). Lung cancer exhibited the fewest uniquely upregulated genes, whereas endometrial cancer showed the fewest unique downregulated genes. Notably, lung cancer had a global trend toward downregulation, whereas endometrial cancer displayed more upregulated genes. Despite some overlap, most differentially expressed genes were unique to each tumor type, reinforcing the cancer-specific nature of transcriptomic dysregulation.

### 3.4 Validation of case study results and evaluation of analytical efficiency

To confirm the reliability of the analytical framework, we compared the results obtained in both case studies with findings reported in the original publications. Across proteomics and transcriptomics analyses, key biomarkers, differential expression patterns and co-expression signatures were consistently reproduced. In the proteomics case study, all four highlighted proteins (*TCL1A*, *TNFRSF9*, *CD22*, and *FCRL3*) exhibited the same directionality and biological relevance (Álvez *et al.* 2023). Similarly, in the transcriptomics case study, major tumor-associated genes such as *CA9*, *SPP1*, and *PRAME* demonstrated expression differences concordant with previous literature, validating that HDAnalyzeR accurately captures disease-specific molecular phenotypes.

To assess analytical efficiency, we benchmarked HDAnalyzeR against equivalent manual workflows replicating the same analyses (Table 2, available as supplementary data at *Bioinformatics Advances* online). The comparison covers computational complexity, including package dependencies, total lines of code, number of function calls and runtime, and highlights a substantial reduction in user burden. HDAnalyzeR consistently required fewer dependencies and 50%–80% fewer lines of code, while runtimes remained essentially unchanged due to the underlying algorithms being identical.

## 4 Conclusions

We present a versatile and user-friendly R package designed to support gene and protein expression analyses across a wide range of platforms and study designs. The package promotes good practices in data handling and analysis, offering modular workflows for quality control, exploratory data analysis, biomarker discovery, and beyond. With flexibility for both standard and customized analyses, the tool accommodates users with varying levels of expertise. Comprehensive documentation, interactive tutorials, and ready-to-use visualizations ensure proper interpretation of results, making it a plug-and-play solution for reproducible, publication-quality research.

In the first use case, focused on distinguishing CLL from other hematological malignancies using high-dimensional proteomics data, the pipeline identified a biologically relevant protein module (*n* = 117) strongly associated with the CLL phenotype. Through integrated WGCNA, enrichment, differential expression and LASSO modeling, a minimal protein panel of eight features was derived, achieving perfect classification performance (AUC = 1.0) on a held-out validation set. In the second case study, we identified various unique and overlapping differentially expressed genes, using transcriptomic data from CPTAC solid tumor cohorts. Furthermore, dimensionality reduction using PCA highlighted transcriptome-level separation between tumor and control samples across all three cancer types. The top scoring genes in both analyses aligned with prior literature, further confirming the biological validity of the outputs. Overall, the use of HDAnalyzeR reduced the required codebase by over 80% in some cases and shortened the total analysis time from multiple days to a few hours.

## Supplementary Material

vbag020_Supplementary_Data

## Data Availability

All code necessary for the data analysis and visualization presented in the Case Studies is available at: https://github.com/kantonopoulos/HDAnalyzeR/tree/main/case_studies. The normalized U-CAN proteomics dataset used in Olink Case Study can be found in the BioStudies database and is available under the accession code S-BSST935 (Álvez *et al.* 2023).
