# Supplementary material for: HDAnalyzeR: streamlining data analysis for biomarker research
Source: Bioinform Adv. 2026 Jan 23;6(1):vbag020. doi: 10.1093/bioadv/vbag020 (PMC12925248; doi:10.1093/bioadv/vbag020)
Supplement: vbag020_Supplementary_Data [file vbag020_supplementary_data.zip › supplementary.pdf]

HDAnalyzeR: Streamlining Data Analysis for Biomarker Research – Supplementary Material

Konstantinos Antonopoulos[1], Emil Johansson[1], Josefin Kenrick[1], Leo Dahl[1], Fredrik Edfors[1], Mathias Uhlén[1,2,*], María Bueno Álvez[1,*]

[1]Department of Protein Science, SciLifeLab, KTH Royal Institute of Technology, Stockholm, Sweden., [2]Department of Neuroscience, Karolinska Institutet, Stockholm, Sweden.

*To whom correspondence should be addressed.

Supplementary Table 1. Comparative overview of commonly used R/Bioconductor packages for proteomics and transcriptomics analysis across key analytical domains.

Package	Imputation	Normalization	Dimensionality Reduction	Protein Co-expression	Differential Expression	Machine Learning	Pathway Enrichment	Visualization
HDAnalyzeR	✅	✅	✅	✅	✅	✅	✅	✅
HDAnalyzeR-app	✅		✅		✅	✅		✅
caret						✅		
clusterProfiler							✅	
DEP	✅	✅	✅		✅			✅
DESeq2		✅	✅		✅			
edgeR		✅			✅			
fgsea							✅	
limma		✅			✅			
missForest	✅							
mixOmics			✅			✅		✅
msImpute	✅							
MSnbase		✅						
MSqRob2					✅			
MSstats		✅			✅			
PCAtools			✅					✅
proDA					✅			
prolfqua		✅			✅			
prolfquapp		✅			✅			
proteomicsLFQ		✅						
randomForest						✅		
scater			✅					✅
Seurat			✅			✅		✅
ComBat		✅						
tidymodels	✅	✅				✅		
WGCNA				✅				

Supplementary Table 2. Comparison of analytical efficiency between HDAnalyzeR and equivalent manual workflows.

Case Study	Workflow	Imported Packages	Lines of Code	Function Calls	Runtime (s)	Validated Outcomes
Proteomics	HDAnalyzeR	4	88	55	52	YES
	Manual	26	459	373	55	YES
Transcriptomics	HDAnalyzeR	8	296	133	141	YES
	Manual	15	558	480	142	YES

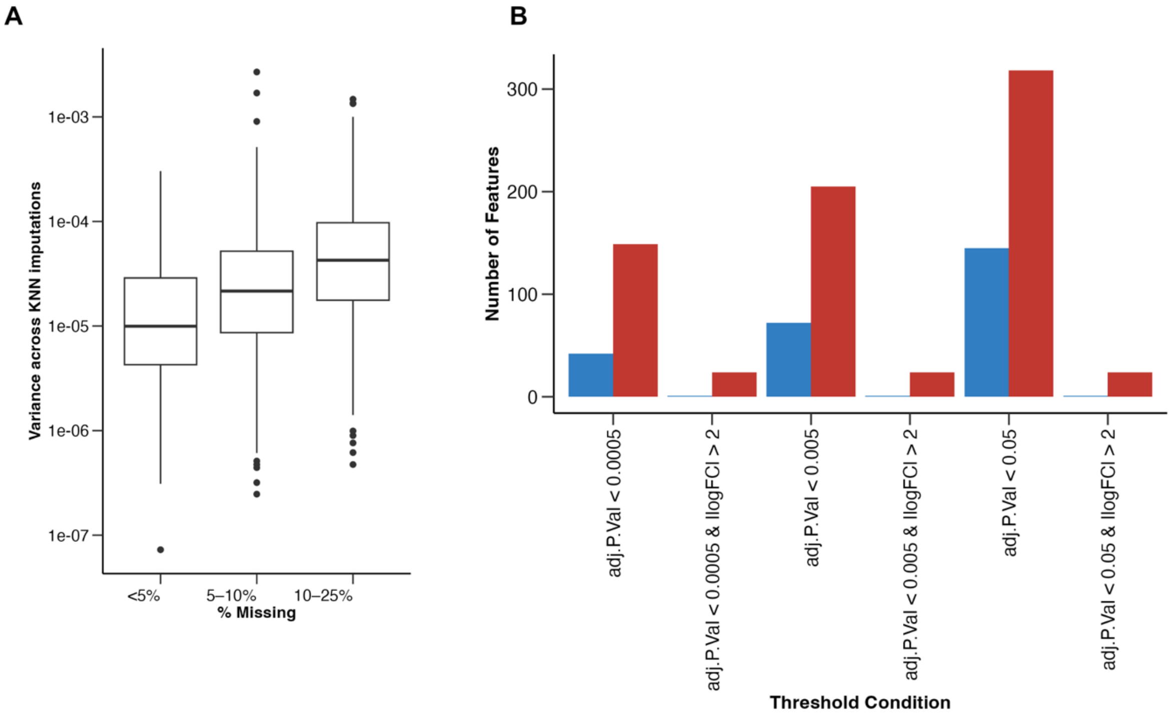

Supplementary Figure 1. Hyperparameter sensitivity analysis results for A. KNN-imputation k and **B.** adjusted p-value and log(Fold Change) cutoffs in differential expression analysis.
